# Computational Micromodel for Epigenetic Mechanisms

**DOI:** 10.1371/journal.pone.0014031

**Published:** 2010-11-30

**Authors:** Karthika Raghavan, Heather J. Ruskin, Dimitri Perrin, Francois Goasmat, John Burns

**Affiliations:** 1 Centre for Scientific Computing and Complex Systems Modeling, Dublin City University, Dublin, Ireland; 2 Institute of Technology, Tallaght, Ireland; University of Barcelona, Spain

## Abstract

Characterization of the epigenetic profile of humans since the initial breakthrough on the human genome project has strongly established the key role of histone modifications and DNA methylation. These dynamic elements interact to determine the normal level of expression or methylation status of the constituent genes in the genome. Recently, considerable evidence has been put forward to demonstrate that environmental stress implicitly alters epigenetic patterns causing imbalance that can lead to cancer initiation. This chain of consequences has motivated attempts to computationally model the influence of histone modification and DNA methylation in gene expression and investigate their intrinsic interdependency. In this paper, we explore the relation between DNA methylation and transcription and characterize in detail the histone modifications for specific DNA methylation levels using a stochastic approach.

## Introduction

The term *Epigenetics* (referring to any phenotypic changes caused by non-mutational factors), was introduced by Waddington in 1940 [1]. The epigenetic layer present in living organisms controls the expression of genes within the genome. Earlier research [2] concentrated on the regions of *Heterochromatin* (densely packed regions within the genome that correspond to the *least actively* expressed genes) and *Euchromatin* (less densely packed regions within the nucleus that contain *most expressed* genes), but subsequently other factors in epigenetics such as *DNA Methylation*, *Histone Modifications* and other assistive proteins such as the *Polycomb* (facilitate alteration of chromatin structure affecting gene expression) were identified [3], [4]. Histones are octomeric core proteins that protect DNA, from restriction enzymes and also act as bolsters in chromatin condensation [5]. The huge amount of genetic information in DNA requires structural condensation inside the nucleus, hence the DNA strand is wound around these histone proteins, forming a unit of *Nucleosome*. Several of these nucleosomes, on further compression are densely assembled to form a chromatin unit. Based on the density of packing, the chromatin form regions of euchromatin or heterochromatin.

Within each human genome, the control over the gene expression is carried out through a well-established co-ordination between DNA “methylation” and Histone “modifications” [6]. DNA methylation refers to the modification of DNA by addition of a methyl group to the cytosine base and is the most stable, heritable and well conserved epigenetic change, introduced and maintained [7], [8] by a family of enzymes called DNA Methyl Transferases (DNMT) [9]. The histone octomer contains two sets of four types (H2-A and H2-B) and (H3 and H4) of histones that pair with one another respectively and a 5th type H1 that binds DNA to the histone for chromatin condensation [6]. A combination of modifications (such as acetylation, methylation, phosphorylation, ubiquitination and sumoylation), within specific amino acids in each histone type leads to gene expression or inactivation [10]. It has also been found recently, that these histone modification patterns, unlike DNA methylation, are dynamic in nature and are completely recreated during DNA replication, transcription and cell division [6].

We do not know precisely what patterns are set during gene expression and how these stimulate transcription by activating or deactivating certain factors. However, work has been reported on the significance of individual histone modifications [11], [12]. In fact, current data reflects the presence of H3 lysine 4 (H3 K4) methylation during transcription [12]. The literature also suggests that activation of one change leads to successive modifications of other amino acids [13]. It is well known that the global acetylation of histones is higher during gene transcription whereas global methylation is more likely during suppression of transcription [6], [12]. Even though new findings with regard to the impact of several histone modifications have been reported, inconsistency of precise information with regard to histone modification for a particular event is a major challenge. It is also not known how the histone modifications are orchestrated under high DNA methylation or when there is high transcription. Despite this insufficiency, we do know for sure that the interactions between histones and DNA methylation are disrupted at some stage, during the onset of cancer. An abnormal epigenetic scenario is observed in cancer cells, which can be attributed to the dysfunction of DNA methylation mechanisms (e.g. conditions of genes being Hyper or Hypo Methylated) [14]. Identification of specific factors has contributed to these circumstances but the picture is incomplete. Hence, apart from aiding in investigation of histone modification, a computational model that mimics the working of epigenetic mechanisms should improve understanding of events leading to malignancy.

## Methods

**Figure 1:**
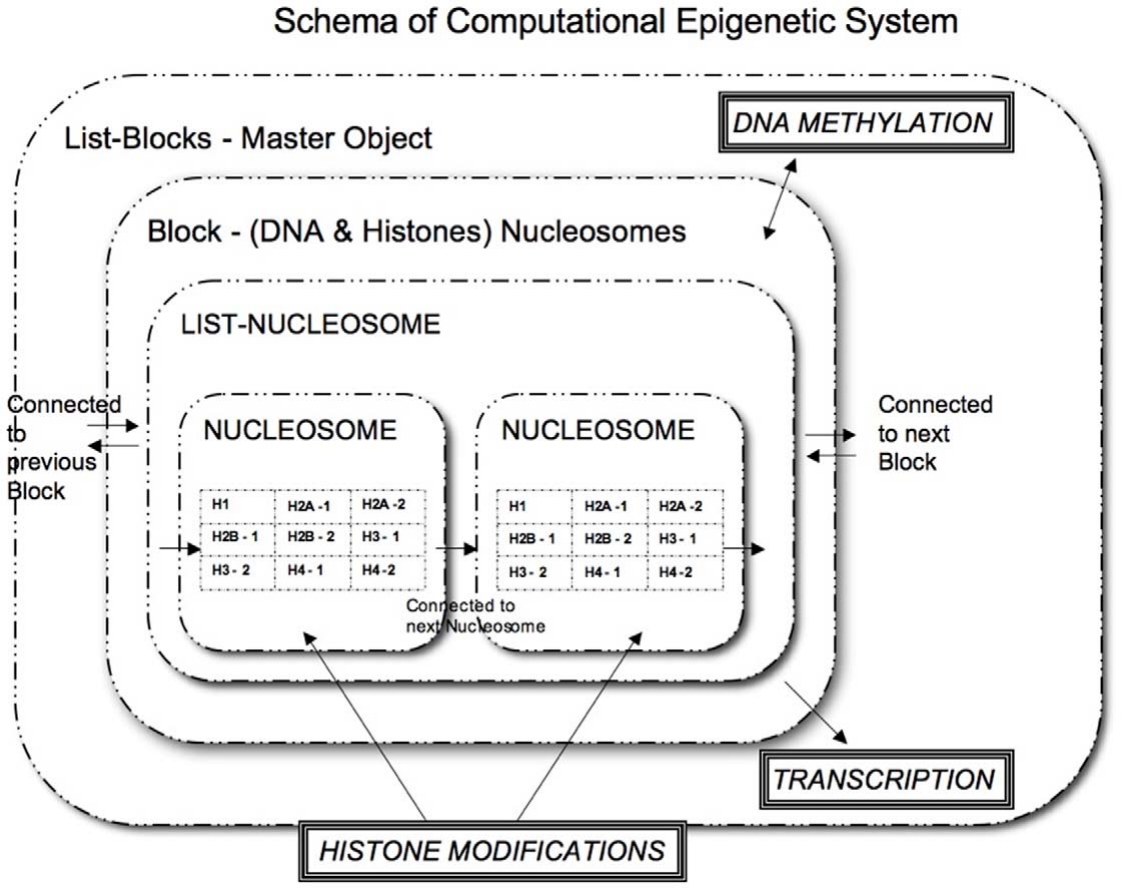
Schema of the Computational Epigenetic Micro-model. Structure and layers of our computational model closely represent known epigenetic mechanisms. The master Object is List-Block which generates a Block of genes. Contained inside each Block are the DNA and Histone objects forming Nucleosome unit. There are 8 histone objects - pairs of H2A,H2B,H3 and H4 along with one H1 object. Each of the histone objects are updated with the modifications over each time-step during the simulation. Hence during each time step, the model is aware of the Histone modifications and DNA Methylation which defines the system evolution.

Our goal is to build a computational model based on inter-relations between epigenetic elements, in order to understand natural system behaviour under abnormal conditions, which may lead to disease onset [15]. This calls for simplified abstraction and incorporation of individual epigenetic events and their inter-dependencies to predict the behaviour of histone evolution under stipulated and controlled conditions. We start by defining hierarchical layers of objects that resemble the natural system. These objects contain attributes that are constantly updated through dynamic changes redefining the objects current state after each time step. Information in the form of possible changes or modifications along with a probability of shift between the changes is introduced before the model execution. Figure 1 displays a simplified construction of our model. One has to keep in mind that the status of epigenetic profile in the model is defined by DNA *Methylation* and Histone *Modifications* and not the DNA and Histone objects themselves.

### Conceptualization

In our model, each object represents a natural entity (such as histone, Nucleosome, Gene Block) in the epigenetic layer. Consequently, the model execution starts with a a master object that generates a chain of gene Blocks. Each gene Block has access to its own set of DNA sequences and Histone objects (forming a Nucleosome Unit). When it comes to Histone objects, each has a set of tables, updated constantly in terms of the chemical modifications that appear after each time step. Although the objects provide a good mimic of the natural system construction, our major focus is on simulating epigenetic events. This is done by allowing the model to move between possible histone states, (containing one combination of possible chemical modifications at a time) over several time-steps, (explained in detail in the next section) using a stochastic approach. This method as a result is used to define the interdependencies between histone modifications, DNA methylation and transcription progress as closely to the natural system as possible.

### Evolution of Histone Modifications

To observe how modifications are handled dynamically, in nature, information (extracted from literature [10]) on the number and type of amino acids for each histone type is fed into the model before the simulation. So when a given type of modification occurs during a particular time step, the corresponding table is updated within that Histone. This encoded information is used to define the intrinsic interdependencies of Histone Modifications, how these affect and are affected by the level of DNA Methylation and their combined effect on the output parameter “Transcription”.

### Data Collection and Representation

**Figure 2:**
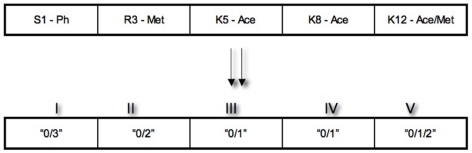
General representation of histone *states* in our model. The number of modifiable amino acids chosen for each histone type differs. In general, each modification is encoded as a number - Acetylation as “1”, Methylation as “2”, Phosphorylation as “3” and no modifications as “0”. The string of numbers or the current Histone *state* represents the possible combination of modifications within that particular histone type.

Table 1 gives the details of the number of amino acids, their positions (selected from the population of amino acids that form the histones), the corresponding modification types and the possible number of histone states generated based on the information obtained from literature [3], [6], [10]–[12]. This information is stored in the model during each simulation as a set of conditions, which must be satisfied (i.e. possible combinations of histone modifications that exist in nature). To represent the amino acid modifications in histones more conveniently, each chemical modification is encoded as a number, (Acetylation as “1”, Methylation as “2”, Phosphorylation as “3” and no modifications as “0”). In the case of the H4 type histone (as shown in Figure 2), an example of H4 histone “state” has only 5 amino acids and each amino acid has a particular modification associated with it. If the current combination of modifications or (*histone state*) is “3-0-0-0-0” it can be interpreted as, the first amino acid (S1) is phosphorylated and the other amino acids (Table 1) are not modified. This process generates a large combination of the possible states in each histone type.

**Table 1:** Amino acid positions and modifications.

S.No.	Histone Type	No. of Amino acids	Amino Acid & Position	Corresponding Modification	No. of States
1.	H1	0	-	-	-
2.	H2A	4	S1-R3-K5-K9	Ph-Met-Ace-Ace	16
3.	H2B	10	K5-S10-K11-K12-S14-K15-K16-K20-K23-K24	Ace/Met-Ph-Ace-Ace-Ph-Ace-Ace-Ace-Met-Ace	1536
4.	*H3	6	R2-T3-K4-R8-K9-S10-T11-K14-R17-K18-T22-K23-R26-K27-S28-T32-K36-K37	Met-Ph-Met-Met-Ace/Met-Ph-Ph-Ace/Met-Met-Ace/Met-Ph-Ace/Met-Met-Ace/Met-Ph-Ph-Ace/Met-Met	6300
5.	H4	5	S1-R3-K5-K8-K12	Ph-Met-Ace-Ace-Ace/Met	48

Details of specific amino acids and their corresponding modifications in all histone types.

*- H3 has a special type of representation based on amino acid type and the corresponding modification. K - Lysine, S - Serine, T - Threonine, R - Arginine, Ace - Acetylation, Met - Methylation, Ph - Phosphorylation.

**Figure 3:**
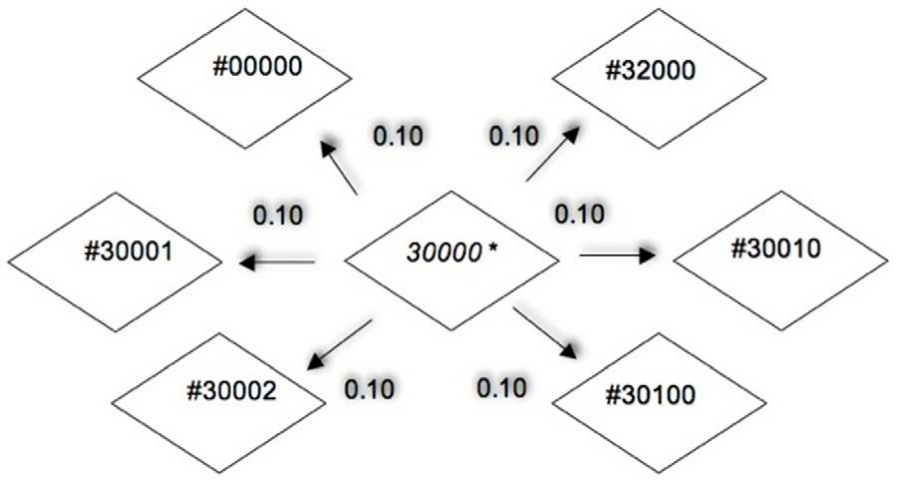
Probability of shift between a histone state and its neighbour. Only one change is possible at each time step hence each histone state can potentially shift to only one of its specific neighbours. * = Current state. # - neighboring state. Probabilities of shift (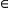
[0,1]) can be given by the user initially.

Each time-step or *Iteration* of the model corresponds to addition or removal of a modification group from the possible combination of histone states. Equivalently this step resembles the action of enzymes which are involved in chemical modification of histone proteins. In the computational model, only one change or modification is made at each iteration when the model moves between the possible histone states, based on probability of shifts. The potential shift to a “neighbouring state” from the current histone state is calculated during each iteration of the model. Probabilities of shift also provide a window of control to introduce stress to the system so as to see how the output parameters and the modifications fluctuate over several time-steps. When there is a shift between states, based on the given probability, the corresponding modification graph in each histone type is updated with the changes. In this way, the model can keep track of the dynamic changes easily and use these to describe the resulting output parameters. Our model can also handle multiple additions of the same modification in an amino acid (Mono/di/tri acetylation, methylation or phosphorylation [10]). Although this is invisible to the user, it is taken into account during calculation of global modification levels in each nucleosome. The actual transition that occurs between possible histone states is decided **randomly**, unless the user wishes to input a revised probability distribution (i.e. based on known or desired experiments). This random function, which decides the next state, is based on a uniform distribution, and returns the index of next random state chosen. If further input by user is necessary during the model run, the probable path to achieve the user desired histone state for a specific time step is calculated by Dijkstra algorithm [16]. The algorithm assumes each histone state to be a node and its probability of shift to a neighboring state as an edge. (Figure 3)

### H3 Modification

The possible number of amino acid modifications for H3 histone obtained from literature was prohibitively large. In consequence these are stored in a different manner to permit compression. A one dimensional array of size *six*, based on the importance of six specific types of modifications and their corresponding amino acids is considered. For example, given a coded representation “4-0-0-0-0-0” the first position corresponds to all Arginines that could be methylated (see Table 2). This allows the system to choose and modify any arginine from its population. i.e. this could be one among R2/R8/R17/R26 or all of them together. A value “V” from the closed range [0,4] is chosen randomly to show the number of arginines modified (based on a uniform distribution random function that returns a random amino acid and the number to be modified in each array position). In the H3 array,while the first position corresponds to methylation of any or all arginines (V 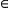 [0,4]), second position to threonine phosphorylation (V 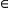 [0,4]) and the third to serine phosphorylations (V 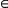 [0,4]), [6], [10], [12], the fourth position in particular corresponds to lysine methylation (V 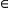 [0,2]) that could relate to high transcription. In contrast, the fifth position relates to lysine methylation (V 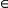 [0,6]) that potentially encourages more DNA methylation and position 6 (V 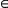 [0,6]) represents acetylation modifications that appear during transcription. As a side effect of compression, the user cannot choose any specific amino (such as R2 or R8 etc) to be modified during the iterations since the model deliberately permits random choice. The details on grouping of amino acids and the compression is given in Table 2.

**Table 2:** Compression of H3 type histone.

Position in Compressed Array	No. of Amino acids	Corresponding Modification	Amino Acids in H3 Modified
1.	4	Methylation	R2,R8,R17,R26
2.	4	Phosphorylation	T3,T11,T22,T32
3.	2	Phosphorylation	S10,S28
4.	2	Methylation	K4,K37
5.	6	Methylation	K9, K14, K18, K23, K27, K36
6.	6	Acetylation	K9, K14, K18, K23, K27, K36

Details on the Compression of H3 histone states. Content of amino acids is classified based on the significance of amino acid type and modification that applies, into 6 groups. K - Lysine, S - Serine, T - Threonine, R - Arginine.

### Epigenetic Interdependency

Our system has a simple yet strong and well defined inter-dependency between histone evolution, transcription rate and level of DNA methylation inside each Block. There are 3 main interactions in our model.

a. Histone Modifications
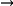
Transcription.

Equations 1(a), 1(b) and 1(c) define how Transcription (T) is calculated after each time interval in our model. The user can set the time-interval to 1, 5, 25 or 100 time-steps, since the biological rate of change of all types of modifications is elusive. Here, the Transcription variable is affected by the number of modifications in all nucleosomes in a Block. The choice of an exponential function in any application, is based on expressing an output that depends on variables that are continuously changing. In our case, the histone modifications are dynamic and used to define the instantaneous state of the model at any time point of the simulation.
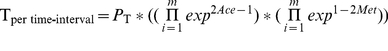
‐(1(a))


m = No of time-steps set by the user in a time-interval

P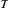 = Probability of Transcription occurring (by default this value is set to 50% or 0.5 – unbiased)



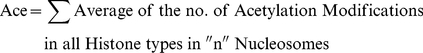
‐(1(b))

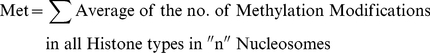
‐(1(c))Here the probability of Transcription to occur is 50% (or 0.50), which is altered by the Histone modifications, hence making the Transcription event a function of the modifications within this stochastic model. Also, the system ensures that if a promoter type Block has high levels of DNA methylation, transcription is blocked for all the gene Blocks that follow the promoter, in agreement with the literature [3]. This step is implemented so that only the promoter decides transcription of the genes, as occurs in nature [6].

The second interaction is:

b. Histone Modifications 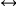 DNA Methylation.

Based on information from literature, the system allows H3 and H4 type histones alone to influence DNA methylation and vice versa.

The influence of DNA methylation on direction of histone evolution is as follows,

(i) Probability Values for histone states containing more Acetylation Modification –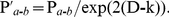
‐(2(a))


(ii) Probability Values for histone states containing more Methylation Modification –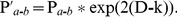
‐(2(b))

k = Mean DNA Methylation Value (set to 50% or 0.50)
a, b = current and neighbouring histone states (H3 and H4 types) respectively.
P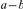 = Initial Probability of shift from state a to state b.
P′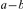 = Probability of shift from state a to state b in the successive iteration.
D = DNA Methylation level in initial iteration.

System ensures that probability values are maintained within the range of 0 and 1 with the help of a *scaling factor* or the mean DNA Methylation value. During each time-step, probability of shift of every histone state (H3 and H4), is altered by DNA Methylation level (as given in equations 2(a) and 2(b)). The user must set the initial probability for the first iteration.

Conversely, histone states (acetylation and methylation modifications) can be used to express the level of DNA methylation, which is calculated in two ways at the start of each iteration.

1. The user can specify a value between 0 and 1, else
2. Based on the initial Histone states chosen, the system calculates the DNA methylation value.

DNA methylation for one Block is calculated in a very simple way in the model.
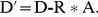
‐(3(a))

D′ = DNA methylation for current Iteration.
D = DNA methylation from previous Iteration.
R = Random Value (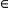
[0,1.0]) generated by the system and based on the uniform distribution.
A = Average of the ratios of the current level to the maximum level possible in methylation and acetylation modification in a Block.

The DNA methylation for the first iteration, if not provided by the user, assumes the value of A. The model utilizes a random value (generated from the uniform distribution) to induce a stochastic behaviour inside the model and also connect the interactions of histone modifications with DNA methylation. The formula 3(a) is implemented within the system (for successive iterations) based on a *conditional probability*. The system generates another random value (based on uniform distribution) and if this value is less than 5% of DNA methylation value (from previous iteration), the formula is implemented. This threshold step is very important since it controls the system evolution and does not allow all modifications to have a uniform effect on DNA methylation. One has to note that the transcription rate is calculated based on the time-interval set by the user and DNA methylation values are set after each iteration or time-step. The third type of interaction, a consequence of the two mentioned above, is discussed in the result section. Hence through these interdependencies in a simple manner, we try to mimic the mechanisms that control gene expression.

### Simulation Process

The steps given below explain the simulation in a simple and concise manner.

1. *Read and Store Inputs*
  - Histone Data -The possible combinations of Histone modification as described above are read and stored in the model. These include *string of histone states* and the *probabilities of shift* between the states. (The possible types of modifications are given in Tables 1 and 2)
  - User Selected Values are provided –
    - Default Parameters: Number of Blocks, number of nucleosomes per Block and total number of iterations(or time-steps) and time-intervals. (Figure 1)
    - Optional Parameters: DNA methylation and histone states preferred by the user (in which Block, nucleosome and at what iteration/time-step)
2. *Create Objects*
  - Based on the number specified, as many objects are created – Blocks (promoters/genes/ isolator/Introns/silencer), nucleosomes, nine histone types (default) and modification tables for each histone.
3. *Simulate*
  - If the user has not chosen to explore a preferred histone state, start with zero modifications. Based on the DNA Methylation value (either mentioned by user or calculated based on those histone states in the current iteration), and the probabilities of shift for each state, choose random states for the next iteration. Simultaneously update the modification tables based on the current state. For example if state 02002 in H4 is chosen, update Methylation tables for H4 histone.
  - For specific time-intervals, record the transcription rate (using equations 1(a),(b) and (c)), and after each time-step calculate the DNA methylation value (based on the modification tables Ð calculated as mentioned above or by taking the value specified by the user in a desired time-step). Also, alter the probabilities of shift based on the DNA value from previous time-step. (using formula (2(a) and (b))
  - Continue till maximum value of iteration is reached.
4. *Store Outputs*
  - Results for the specified time interval, inside each Block –
    - Transcription rate
    - DNA Methylation level
    - Global Modification levels for each Block (Methylation, Phosphorylation and Acetylation)
    - Count of the number of times each state is visited in all 8 histones for each nucleosome.

### Assumptions

As the major focus is on histone evolution,we make a few simplifications here to test the system reliability.

1. The model currently handles only three modifications i.e. Acetylation, Methylation and Phosphorylation as their biological significance is known from literature [10].
2. Although our model can handle several hundred nucleosomes per Block (as in reality), we illustrate with only one nucleosome per Block to track and analyze the evolution of histones over several time-steps. This ensures that the evolution of modifications in a single nucleosome is clearly identified and changes are demonstrated. The nucleosome number will be increased for further investigations to improve realism.
3. Our System is initialised with “zero” modifications, which are slowly increased over several iterations.(Intervention by the user to permit input of desired histone states in any of the time steps is currently not allowed.)
4. DNA Methylation during the first iteration for the promoter Block was specified by the user (such as high and low conditions of DNA methylation values as specified in results section). For subsequent simulation, the system evolution determines the values.

## Results and Discussion

In order to investigate the system behaviour, 64 objects of Block type with a single nucleosome per Block was implemented, and evolution was observed over 5000 iterations. Sixteen promoters, each of which controlled 3 subsequent genes, were added within the chain, to form 64 Blocks. Histone states, transcription progression, DNA methylation and global histone modification levels for every Block were recorded every 25 iterations.

### Transcription Progression

The third type of interaction in our model whose relation is analyzed below is,

c. DNA Methylation 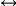 Transcription.

**Figure 4:**
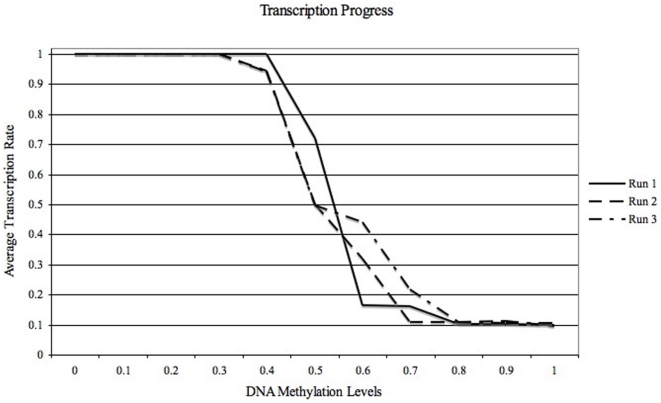
Average Transcription Progression derived for 16 Promoters over 5000 iterations during 3 different simulation runs. The third type of interaction between Transcription rate and DNA methylation level (or percentage) was observed here. Transcription rate (25 time-steps = 1 time-interval) is inversely proportional to DNA methylation level (decided by user in this case, for testing purposes).

As an effect of the first two interactions mentioned above in methods section, the model is able to efficiently simulate an inverse relation between the components of the third interaction, *Transcription(T)* and *DNA Methylation* as reported in the literature. Transcription values (T
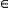
[0,1.0], represented in Figure 4), for increasing DNA methylation levels (specified by the user, in this case) were observed during 3 different simulation runs. Figure 4 depicts the relation and effect of Transcription on DNA Methylation and vice versa. Here, an inverse relation between transcription and DNA methylation levels is consistently prominent. Higher transcription is observed when the DNA methylation is 0.35 or less, while higher DNA methylation values, (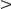
0.75) evidently prevent any increase in transcription.This behaviour is a reflection of the model in choosing specific histone modification over several iterations. For DNA methylation in the range 0.47 to 0.7, (hemi-methylation state), the rate of transcription is severely affected. We believe that this observation could co-relate to how transcription is blocked under methylation of the CpG islands within the promoter. These findings, for the simplified model, are broadly in agreement with the real system as reported in the literature [6].

### Histone Evolution

Here, we analyse histone modifications for only two cases, i.e. *high* DNA methylation (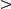
0.85) and *low* DNA methylation (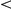
0.15), solely for the *Promoter* type Block as any changes to this Block affect the succeeding genes. These conditions are analyzed to study biological cases such as, those which apply when an unexpressed oncogene is activated or when a tumor suppressor gene is inactivated.

### Case 1: Histone Evolution during low DNA Methylation

For small fixed levels of DNA methylation, (refer to Figure 4) acetylated histone states are preferentially chosen, which in turn lead to a stable and high transcription rate. These simulations are carried out to show how the system effectively emulates the biological process of transcription of genes for low DNA methylation levels. Considerable evidence from literature show that histone types H3 and H4 are significant during transcription and their signatures, (or constituent amino acid modifications), determine DNA methylation and transcription levels, [6], [13], [17], [18]. Hence we focus on tracing these evolution types alone in our model.

**Figure 5:**
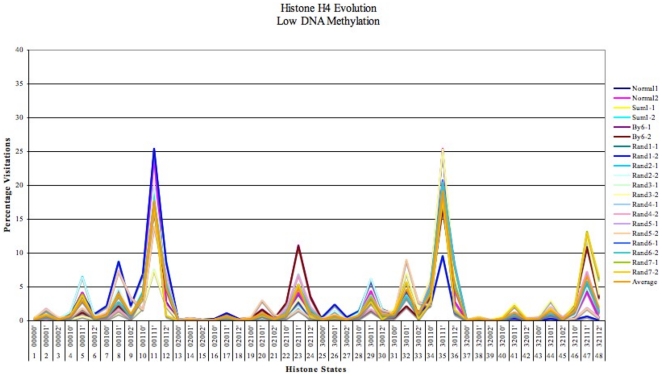
Evolution of H4 (H4-1 and H4-2) histone states in the 16 promoters for 10 different datasets during low DNA methylation levels (<0.15 or 15%). H4-1 and H4-2 histone states were tested with 10 datasets of random probability values (represented by colors in the graph).

Figure 5 shows the average percentage preference in 16 promoters, of all possible states in H4 histone for 10 datasets. We tested the consistency and robustness of the system by initially assigning 10 datasets with various “probabilities of shift” for H4 (H4-1 and H4-2) type histone. These probabilities (of a move from the current state to any of its neighbors) were generated randomly by a system defined function (based on a pseudo random number generator - Mersenne Twister, which is robust, has a large range of period and a high order of dimensional equidistribution [19]). Acetylated amino acids states, such as the 11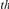, 35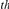 and 47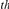 predominated in more than 75% of the datasets i.e. states containing acetylated amino acids such as K5, K8 and K12 (see Table 1) were highly visited. Even when the probability for one of the three preferred states was lowered during any test set, the system preferred the other two states containing lysine acetylation. Such consistent results demonstrate the ability of our model to reproduce the presence of these modifications during transcription, (as reported [20], [21] in particular, during expression of oncogenes).

#### H3 Analysis

**Figure 6:**
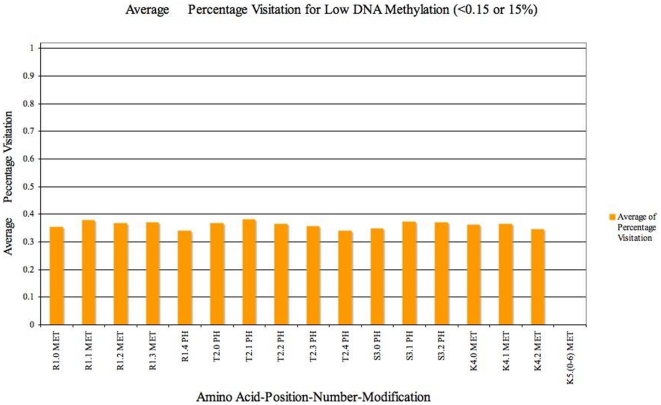
Analysis of Average percentage visits of H3 histone states containing *Lysine Acetylation .i.e K6* in 16 promoters after 5000 iterations (for low levels of DNA Methylation (<0.15 or 15%)). States containing Lysine acetylation are visited the most. Hence we analyse the the average of percentage visitation of model to all other modifications (except Lysine Acetylation - K6 in Figure) during the simulation. Here each unit in the X-axis represents an amino acid–position in H3 array–number of Amino acids–Modification possible. The Y axis elaborates on the average percentage visitation of H3 states that contain the modification depicted in each unit of X axis.

**Figure 7:**
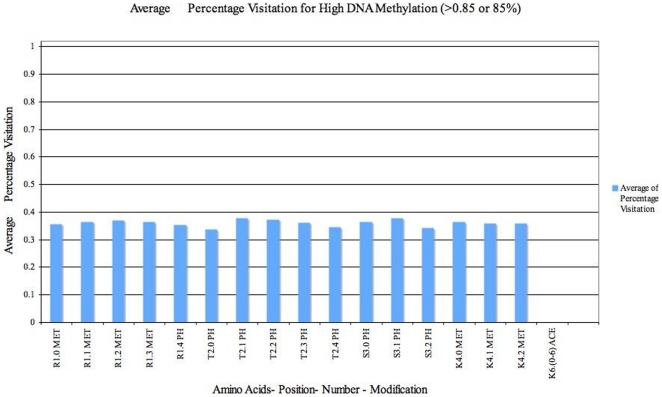
Analysis of Average percentage visits of H3 histone states containing *Lysine Methylation i.e. K5* in 16 promoters after 5000 iterations (for high levels of DNA Methylation (<0.85 or 85%)). States containing Lysine Methylation are visited the most. Hence we analyse the average of percentage visitation of model to all other modifications (except Lysine Methylation K5) during the simulation. Each unit in the X-axis represents an amino acid–position in H3 array–number of Amino acids changeable–Modification. The Y axis elaborates on the average percentage visitation of H3 states that contain the modification given on the X axis.

The depiction and interpretation of H3 results reflect the way these are addressed in the model. In each of the H3 associated figures, (Figures 6 and 7), a unit on the X-axis represents an expansion of what the linear array of H3 histone stands for (Amino acid, Position in H3 Array, Number, Modification). The Y-axis gives an average percentage of visitation of the states containing the modifications described by each unit in the X-axis. Since the number of H3 histone states generated, even after compression, is the largest among all histone types (refer to Table 1), we report and analyze specific and prominent H3 states that are significant based on the information from literature.

H3 histone states that contain maximum lysine acetylation (refer Table 2) such as K6 are only visited during high level of transcription. Hence, we analyse the modifications within those states in particular. These states, contain least or no Lysine methylation, (corresponding to position K5(0–6) in Figure 6 and in H3 array- of amino acids K9, K14, K18, K23, K27, K36 ). Also, in these states, phosphorylation of serines is higher, (i.e position 3 in H3 array depicts S10 and S28 phosphorylation - refer to Table 1), as substantiated also by literature [17]. In general, however we find that preference given to other amino acids positions (R1, T2 and K4 series) and their corresponding modifications is very similiar. This means that apart from serine, other modifications could be neutral or default modification during transcription.

### Case 2: Histone Evolution during High DNA Methylation

**Figure 8:**
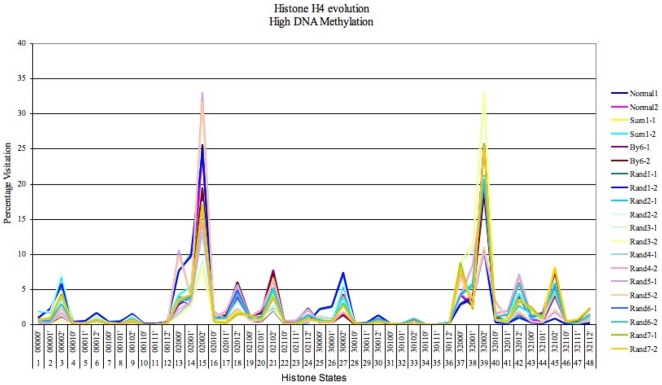
Evolution of H4 (H4-1 and H4-2) histone states in the 16 promoters for 10 different datasets during high DNA methylation levels (<0.85 or 85%). H4-1 and H4-2 histone states were tested with 10 dataset of random probability values (represented by colors in the graph).

For higher levels of DNA methylation (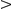
0.85, Figure 8) during the simulation, the preference is more towards choosing methylated histone states. This biased behaviour of the system leads to reduced transcription rate.

Figure 8 shows the average percentage occupation of H4 type histone states for 16 promoters. The system was again tested with 10 datasets with various probabilities assigned to the histone states in H4 (H4-1 and H4-2). The system was found to persistently occupy methylated amino acids, states such as the 15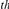, 39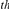 and 45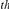 in more than 8 out of 10 datasets i.e. methylation of K12 was predominantly high. Such strong evidence, (during histone deacetylation and methylation) of modification to a crucial lysine position in H4, is a potential indicator of transcription repression and initiation of DNA methylation. Figure 8 hence indicates the possible presence of this modification during real gene repression. Another interesting observation is the appearance of serine phosphorylation (state 39, Figure 8 and state 35, Figure 5) which show the importance of this specific modification during expression or otherwise. This suggests that the modification could be present from the time that the H4 histone complex was formed [22].

#### H3 Analysis

Interpretation of Figure 7 is similar to Figure 6. We analyse specific H3 states so as to aid in comprehension of the results. Figure 7 shows the modifications that were preferred during high DNA Methylation (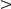
0.85). Only states which contained lysine methylation (amino acid positions such as K9, K14, K18, K23, K27, K36 as in Table 2 - position K5) were visited. Hence we analyse the preference of other modifications within H3 states that contain Lysine methylation. Here, conversely to Figure 6, within those states, lysine acetylation was negligible ( acetylation of K9, K14, K18, K23, K27, K36 - Table 2) as these are preferentially methylated. This is shown by the least number of times the system visited those states that contain Lysine acetylation, (position K6(0–6) in Figure 7). Also, recruitment of states containing high phosphorylated serine was low. Such observations, on the content of lysine acetylations and serine phosphorylations during high DNA methylation suggest that our model can successfully reproduce results from laboratory studies [17] and also indicate presence of other modifications as yet unexplored in the literature.

### Comparative Study

**Figure 9:**
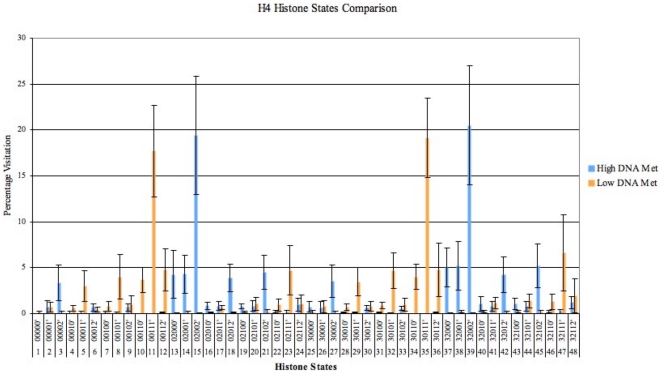
A Comparison between the average (of all 20 test results obtained for H4-1 and H4-2) preferences of H4 states for high and low DNA Methylation Levels. Error bars represent the standard deviation calculated from the total number of visits, for every H4 histone state (occupancy) during the simulation.

Figure 9 contrasts percentage visitation for H4 histone states under high (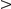
0.85) and low (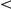
0.15) DNA methylation levels. As DNA methylation controls the direction of histone evolution, the states visited for high levels of DNA methylation are not visited for low levels and vice versa. Standard deviations, shown as error bars, are calculated from the results containing the number of visits for each state. The deviation is high for less visited states and low for highly visited states. This means that the system tolerance to initial selection (determined by random selection using PRNG Mersenne Twister) is also good with specific states consistently chosen over several iterations. This consistency in predicting characteristic histone modifications under defined DNA methylation levels, leverages our models capability to mimic the real system to an accurate level. Hence, we expect to obtain similar Histone patterns under stable DNA methylation values, for corresponding experimental observations.

### Conclusion and Future Work

The current version of the model, has been demonstrated to be capable of reproducing known histone modification under stipulated DNA methylation levels, and also report unexplored modifications such as K12 methylation (Figure 8). Preference of histone states containing Lysine acetylation during high transcription, and increased number of methylation modifications in H3 and H4 states for higher values of DNA Methylation confirms this. Further analysis of the additional modification - (phosphorylation), reveals that for H3 type histone it supports transcription (serine phosphorylation, in Figure 6), while this simultaneously stays neutral in H4 type histone (see Figures 5 and 8). Such results demonstrate the model capability and its potential as a tool to simultaneously trace the evolution of histone modifications for different histone types, and to investigate how the epigenetic profile is affected overall. Stochastic modeling is a powerful method to use when several factors affect the output of a system, with applications across a wide range including the finance sector and social networks amongst others. In our problem, the presence of several histone modifications and their overall dynamic interaction with DNA Methylation, form a complex system with stochastic evolution of constituent elements, thus providing a strong motivation for *in-silico modeling*. While individual results from laboratory experiments in epigenetics and methods to analyze them, have been reported [20]–[22], our model is the first of its kind to determine the occurrence of several modifications at one time-step. This provides a basis for further investigations of abnormal conditions such as Cancer and other genetic disorders. Apart from modifications within H3 and H4 type histone, we are currently investigating H2A and H2B [22] modifications and their influence on the model evolution and output parameters. Also, the model's capability to investigate influence of modifications in one histone type compared to another is being refined. In the long term these studies aim to establish a comprehensive model framework for different histone changes in order to aid in understanding of how successive events can initiate transcription or gene suppression that ultimately influence phenotype of an organism.

While the model described is at an early stage, efforts for improving the model sensitivity to other factors that cause epigenetic changes are in progress. This includes adding more modification types such as Ubiquitination and Sumoylation, if data to support the importance of their contributions are sufficient. While the simple model assigns DNA methylation values based on global histone modifications, calculations based on CG patterns in CpG islands and other regions of the human genome (methylated and non-methylated regions) will add further realism to the study of epigenetic mechanisms. This expansion will accommodate the role and influence of DNA sequences (gene coding and tandem repeat regions), to be taken into account in addition to histone modifications inside the model.
